# Editorial for the *IJMS* Special Issue on “Infection and the Kidney”

**DOI:** 10.3390/ijms24098431

**Published:** 2023-05-08

**Authors:** Takashi Oda

**Affiliations:** Department of Nephrology and Blood Purification, Kidney Disease Center, Tokyo Medical University Hachioji Medical Center, Tokyo 193-0998, Japan; takashio@tokyo-med.ac.jp

The coronavirus disease (COVID-19) pandemic has highlighted the close relationship between infection and kidney injury. Infections induce kidney parenchymal injury directly or indirectly through the various mechanisms of several renal diseases. This Special Issue, entitled “Infection and the Kidney,” focuses on this important and expanding topic of research, providing research and updated reviews on kidney injury observed in association with ongoing or past infection.

Masset et al. [1] described the mechanisms and pathological features of nephropathy associated with various viral infections. Their review comprehensively summarized nearly all viruses that induce kidney injury based on their mechanisms and pathological features, such as acute tubulointerstitial nephritis, acute tubular necrosis, thrombotic microangiopathy (TMA), and glomerulonephritis (membranoproliferative glomerulonephritis, membranous nephropathy, minimal change disease, and collapsing focal segmental glomerulosclerosis). Meanwhile, Teixeira et al. [2] and Vrečko et al. [3] specifically reviewed COVID-19-infection-related kidney injury. Teixeira et al. [2] presented an overview of the overall epidemiology of kidney injury associated with COVID-19 infection (especially acute kidney injury) with original data on the mechanism of viral entry to the kidney (binding with the ACE-2 receptor expressed on the apical side of proximal tubular epithelial cells, entry into the cells via endocytosis, and endosomal acidification). Vrečko et al. [3] focused on the kidney injury of TMA caused by COVID-19 infection. The authors reported that TMA could occur not only during COVID-19 infection at the time of viremia but also after viral clearance, which should be recognized and noted by nephrologists. Among the reported 46 cases of TMA, 18 were thrombotic thrombocytopenic purpura, and the remaining 28 were atypical hemolytic uremic syndrome.

IgA nephropathy (IgAN) is a rare disease for which a link between focal infection and kidney inflammation has been demonstrated in humans, as evidenced by the widespread use and efficacy of tonsillectomy in Japan. Its efficacy has been so great that most centers in Japan have begun using tonsillectomy plus steroid pulse therapy as the standard treatment for IgAN. The developers of this treatment, Hotta et al. [4], contributed a review article describing the relationship between inflammation in the lymphoid tissue of the Waldeyer’s ring (palatine tonsils and epipharyngeal lymphoid tissue) and glomerular lesions causing massive hematuria, also known as glomerular vasculitis. They emphasized the importance of inflammation in this area as the essential focus of focal infection and proposed the so-called “epipharynx–kidney axis” in the development of IgAN. Nagasawa et al. [5] demonstrated the important roles of periodontal bacteria, especially *Porphyromonas gingivalis*, in IgAN by showing a higher presence of these bacteria within the tonsils of patients with IgAN than in those of patients with simple tonsillitis, together with experimental data from a mouse model. Nagasawa et al. [6] also contributed a review article describing the role of oral bacterial species related to dental caries and periodontitis related to IgAN.

Regarding infection-related glomerulonephritis (IRGN), two elegant reviews were included: one written by Yoshizawa et al. [7], the discoverer of a nephritogenic protein for post-streptococcal acute glomerulonephritis (nephritis-associated plasmin receptor: NAPlr), and the other written by Takayasu et al. [8], who established the concept of methicillin-resistant *Staphylococcus aureus* (MRSA)-infection-associated glomerulonephritis. Yoshizawa et al. [7] described the mechanism by which NAPlr, which they identified as a nephritogenic protein for PSAGN, and related plasmin activity were also detected in IRGN caused by bacteria other than *Streptococcus*. Therefore, NAPlr could be used as a general biomarker of bacterial IRGN (Figure 1). Furthermore, they provided unique insight into the mechanisms by which C3 nephropathy-like glomerular lesions without immunoglobulin deposition are often formed in IRGN. Takayasu et al. [8] reported a detailed clinicopathological review of staphylococcal-infection-associated nephritis, which usually presents pathologically as IgA-dominant IRGN. As a mechanism of disease development, staphylococcal enterotoxins with superantigenic properties may induce a marked activation of T cells, an increase in specific variable regions of T-cell-receptor β-chain-positive cells, hypercytokinemia, and increased polyclonal immune complexes.

Finally, Uchida et al. [9] summarized the important roles of innate immune cells, including natural killer T (NKT) and natural killer (NK) cells, with the support of liver macrophages in the development of sepsis-associated acute kidney injury, which is a highly life-threatening condition. Data on murine models of acute kidney injury have been introduced; however, limited reports and data on the association among infections, human CD56+ T cells (the functional counterpart of NKT cells in mice), CD56+ NK cells, and human renal diseases have been reviewed.

A detailed understanding of the mechanisms underlying the relationship between infection and the kidneys is important because it may also lead to the elucidation of the pathogenic mechanism of idiopathic renal diseases. This is an area where knowledge is expanding, and further development is expected. Therefore, a second edition of the Special Issue, “Infection and the Kidney 2.0,” is currently being compiled [10].

## Figures and Tables

**Figure 1:**
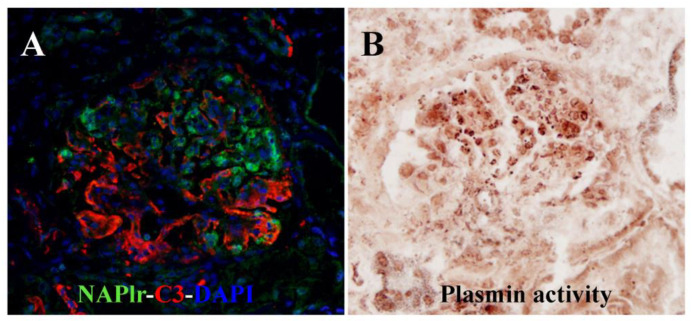
Histological staining for C3, NAPlr, and plasmin activity in the glomeruli of a patient with infection-related glomerulonephritis induced by asymptomatic sinusitis. Double staining for NAPlr (fluorescein isothiocyanate, green) and C3 (Alexa Fluor 594, red) with nuclear staining for DAPI (blue) showed glomerular deposition of NAPlr and C3 (**A**). Glomerular plasmin activity assessed using in situ zymography on a serial section demonstrated a similar distribution as NAPlr deposition, providing histological evidence for the substantial involvement of bacterial infection in the development of glomerulonephritis (**B**). Photographs are modified and cited from the report [7]. NAPlr: nephritis-associated plasmin receptor.
